# Dehydration Stress Contributes to the Enhancement of Plant Defense Response and Mite Performance on Barley

**DOI:** 10.3389/fpls.2018.00458

**Published:** 2018-04-06

**Authors:** M. E. Santamaria, Isabel Diaz, Manuel Martinez

**Affiliations:** ^1^Centro de Biotecnología y Genómica de Plantas, Instituto Nacional de Investigación y Tecnología Agraria y Alimentaria, Universidad Politécnica de Madrid, Madrid, Spain; ^2^Departamento de Biotecnología-Biología Vegetal, Escuela Técnica Superior de Ingeniería Agronómica, Alimentaria y de Biosistemas, Universidad Politécnica de Madrid, Madrid, Spain

**Keywords:** plant-biotic-abiotic interaction, *Hordeum vulgare*, *Tetranychus urticae*, dehydration stress, differential gene expression

## Abstract

Under natural conditions, plants suffer different stresses simultaneously or in a sequential way. At present, the combined effect of biotic and abiotic stressors is one of the most important threats to crop production. Understanding how plants deal with the panoply of potential stresses affecting them is crucial to develop biotechnological tools to protect plants. As well as for drought stress, the economic importance of the spider mite on agriculture is expected to increase due to climate change. Barley is a host of the polyphagous spider mite *Tetranychus urticae* and drought produces important yield losses. To obtain insights on the combined effect of drought and mite stresses on the defensive response of this cereal, we have analyzed the transcriptomic responses of barley plants subjected to dehydration (water-deficit) treatment, spider mite attack, or to the combined dehydration-spider mite stress. The expression patterns of mite-induced responsive genes included many jasmonic acid responsive genes and were quickly induced. In contrast, genes related to dehydration tolerance were later up-regulated. Besides, a higher up-regulation of mite-induced defenses was showed by the combined dehydration and mite treatment than by the individual mite stress. On the other hand, the performance of the mite in dehydration stressed and well-watered plants was tested. Despite the stronger defensive response in plants that suffer dehydration and mite stresses, the spider mite demonstrates a better performance under dehydration condition than in well-watered plants. These results highlight the complexity of the regulatory events leading to the response to a combination of stresses and emphasize the difficulties to predict their consequences on crop production.

## Introduction

One of the most important threats to crop production is the attack of combined biotic and abiotic stressors. Under natural conditions, plants may suffer different stresses simultaneously or in a sequential way. Current knowledge on plant responses to different biotic and abiotic combined stresses has been recently reviewed (Zhang and Sonnewald, 2017). Previous studies have been focused in the impact of simultaneous or sequential exposure to pathogens/pests and abiotic stresses (reviewed in Atkinson and Urwin, 2012; Ramegowda and Senthil-Kumar, 2015). In many cases, exposure of plants to pathogens/pests enhances abiotic stress responses, while abiotic stress weakens disease resistance. Thus, the activation of the responsive mechanisms of the plant is determined not only by the current stress/es, but also by the stress/es suffered previously, and plant response to combined stresses cannot be predicted from the responses to individual stresses (Zhang and Sonnewald, 2017). The understanding of how plants deal with the whole potential stresses affecting them will provide new breakthroughs that could be used in the development of biotechnological tools to protect plants.

At present, drought is one of the most important environmental stresses in agriculture (Fahad et al., 2017). Growing water demand, diminishing water supply and the increasing rainfall variability associated to climate change are expected to increase water deficit impact on crop yield (Lobell et al., 2011). Currently, drought causes yield reductions through negative impacts on plant growth and physiology in several crop species (Barnabás et al., 2008; Daryanto et al., 2016). The plant response to drought includes stomata closure, photosynthesis inhibition, accumulation of osmotically active compounds and protective proteins, changes in the sink/source allocation of several compounds, and triggering of phytohormones-related signaling pathways, mainly abscisic acid (Golldack et al., 2011; Nakashima et al., 2014). These protective mechanisms usually lead to a reduction on crop yield (Farooq et al., 2009; Praba et al., 2009). Besides, *Tetranychus urticae* is a cosmopolitan agricultural acari pest feeding on over 1,100 different plants, many of them of agricultural importance (Migeon and Dorkeld, 2006–2017). Due to high fecundity, inbreeding, and short generation time, Tetranychidae populations quickly develop resistance to acaricides (Van Leeuwen and Dermauw, 2016). Furthermore, increased temperatures and drought stress associated to climate change favor spider mite development. The spider mite life cycle is shortened; more generations are produced per year; and the pest appears earlier and with a broad range of hosts (DeLucia et al., 2012; Ximénez-Embún et al., 2017a). *T. urticae* pierces parenchymatic plant cells using stylets to suck their nutrients, and cause severe chlorosis leading to a reduction in crop yield (Bensoussan et al., 2016). Due to the *T. urticae* impact in agriculture, research on plant responses to spider mite infestation has been performed on crops as well as in model plants (Maserti et al., 2011; Agut et al., 2015; Díaz-Riquelme et al., 2016). Although jasmonic acid is the main hormone involved in the establishment of spider mite-induced defense responses, salicylic acid signaling pathway is also trigger by mite feeding (Ament et al., 2004; Zhurov et al., 2014; Martel et al., 2015; Santamaría et al., 2017).

The effect of drought on the performance of herbivores is uncertain and variable. Meta-analyses have pointed out that water stress tended to decrease the fecundity of chewing and sucking insects, with the strongest negative effects on sap-phloem insects (Koricheva et al., 1998; Huberty and Denno, 2004). In contrast, aphid performance was found to be highest in *Brassica* plants subjected to moderate dehydration stress (Tariq et al., 2013), and the leaf-chewing herbivore *Pieris brassicae* performed better on dehydration-stressed than on well-watered *Alliaria petiolata* plants (Gutbrodt et al., 2011). Regarding herbivore acari, the spider mites *T. urticae* and *T. evansi* appeared to benefit from drought stress in tomato plants because of the improved nutritional value of the leaves (Ximénez-Embún et al., 2016, 2017a). Likewise, the tomato russet mite *Aculops lycopersici* grows faster and causes more damage on drought-stressed tomato plants (Ximénez-Embún et al., 2017b). These data further illustrate the complexity of biotic-abiotic crosstalk under variable environmental conditions and demonstrate potential difficulties in predicting herbivore pest status under changing environments (Foyer et al., 2016).

Based on its wide environmental range, barley (*Hordeum vulgare*) has been proposed as an excellent model for understanding adaptation to climate change (Dawson et al., 2015). Drought is an impact factor for yield losses of this cereal (Wehner et al., 2016). The existence of barley genotypes tolerant and sensitive to drought has been largely used to analyze global gene expression changes after drought stress (Guo et al., 2009; Svoboda et al., 2016; Zeng et al., 2016; Cantalapiedra et al., 2017). Disparities in the results are likely due to differences in experimental set up and plant material (Cantalapiedra et al., 2017). In contrast, transcriptomic analyses on differential gene expression after herbivore attack are scarce. Only transcriptional responses have been characterized upon interaction with aphid species (Delp et al., 2009; Escudero-Martinez et al., 2017) and, as far as we know, no transcriptomic analysis has been performed in barley combining biotic and abiotic stresses.

Barley is a host of the spider mite *T. urticae* (Diaz-Mendoza et al., 2017). As well as for drought stress, the economic importance of the spider mite on agriculture is expected to increase due to climate change. To obtain insights on the combined effect of drought and mite stresses on the defensive response of the plant, in this work we have analyzed the transcriptomic responses of barley plants subjected to dehydration treatment, spider mite attack, or to the combined dehydration-spider mite stress. Besides, the performance of the mite in water stressed and well-watered plants has been analyzed. This knowledge could assist to the development of biotechnological tools for integrated abiotic/biotic management with a practical applicability in the context of climate change.

## Materials and Methods

### Plant Material and Growth Conditions

Grains of the drought-susceptible cultivar “Golden Promise” of barley (*H. vulgare* L.) were germinated in a mixture of soil and vermiculite (3:1) and grown at 23°C under a 16 h light/8 h darkness photoperiod for 7 days in Sanyo MLR-350-H chambers. At this point, plants were subjected to spider mite infestation (M), dehydration stress (D) or the combination of both stresses (DM). Controls (C) were always grown in parallel.

### Spider Mite Material and Growth Conditions

A colony of the two-spotted spider mite *T. urticae* London strain (Acari: Tetranychidae), provided by Dr. Miodrag Grbic (UWO, Canada), was maintained on beans in a Sanyo MLR-350-H growth chamber at 25°C under a 16 h light/8 h darkness photoperiod. This colony was transferred to barley where it was maintained under the same conditions for more than 30 generations to ensure mite adaptation to the host.

### Mite Infestations and Dehydration Treatments

For spider mite infestation, each plant was infested with 20 adult female mites adapted to barley. The infestation was performed by placing a barley leaf with the mites on the experimental plant leaves. A falcon tube with holes was used to help maintaining the leaves together. Barley plants were confined in pots with plastic cylinders covered on top by nylon nets to avoid dispersion of mites. The same isolation system was applied to control and dehydration stressed plants. For dehydration treatments, pots were placed over Petri plastic plates to individualize watering. Plants were subjected to dehydration stress imposed by continuous water deprivation. Controls were watered to maintain soil moisture at 70% (considered as optimal watering). In addition, both stresses were combined at the same time in order to study the effect of mixing biotic and abiotic stresses.

Barley phenotypes were monitored at different time points in control plants and in plants subjected to mites, dehydration or the combination of both stresses. Leaves were harvested after 4, 7, 10, and 13 days. Samples were imaged and scanned, or frozen into liquid nitrogen and stored at -80°C for further analysis. Three independent experiments were performed.

### Physiological and Biochemical Parameters

The effect of spider mite infestation, dehydration and the combination of both stresses was studied 7 days post treatment by determining several physiological parameters, always done at the same time of the day to avoid circadian effects. Aerial plant biomass and soil mass were determined by weighting (Precisa XB 2200 C) at the end of each treatment (fresh weight), and after drying in a stove at 70°C (dry weight). Aerial plant water content (PWC) and soil water content (SWC) were obtained from these measurements (fresh weight minus dry weight). As growth indicators the number of leaves and the length (cm) of the longest leaf were also quantified. In addition, the quantum yield (QY) of the photosystem II and the continuous fluorescence yield (FT) were estimated as indicators of the photosynthesis efficiency using a FluorPen FP 100 (PSI, Drasov, Czechia). Both parameters were measured on the oldest and the youngest leaves. Nine plants from three independent experiments were used per treatment.

### RNA Isolation, cDNA Library Construction and Illumina Sequencing

Total RNA was extracted from frozen barley leaves by the phenol/chloroform method, followed by precipitation with 8 M LiCl (Oñate-Sánchez and Vicente-Carbajosa, 2008) and digested with DNase (Promega). Using poly-T oligo-attached magnetic beads, mRNAs were purified from the total RNA. Then, the mRNAs were fragmented and cDNA was synthesized using random hexamer-primers, DNA polymerase I and RNase H. The double-stranded cDNAs were purified with magnetic beads and ligated to adaptors for Illumina sequencing. The quality and quantity of the library was verified using an Agilent 2100 Bioanalyzer and an ABI StepOnePlus Real-Time PCR system, respectively. The cDNA libraries were sequenced using the Illumina HiSeq2000 platform by the Beijing Genomics Institute (BGI). More than 10M single-end reads were obtained for each sample (three biological replicates). Raw reads in fastq format were firstly filtered and reads with adaptor sequences and low quality reads were removed. RNA-seq data are available on ArrayExpress database at EMBL-EBI^1^ under accession number E-MTAB-6565.

### Sequence Data Analysis and Annotation

The gene and genome sequences of *H. vulgare* retrieved from the PGSB/MIPS PlantsDB website^2^ (Nussbaumer et al., 2013) were used as the reference databases (Mayer et al., 2012). Clean reads were pseudoaligned to the reference High Confidence genes using Kallisto (Bray et al., 2016). The transcript abundance was quantified as TPM (transcripts per million) and 100 bootstrap samples were performed. Differential expressed genes (DEGs) between groups were obtained using the RNA-seq 2G portal^3^. From the 25 methods available in this portal, five methods were selected, which do not use log-transformed data as input and has its own normalization procedure (Zhang et al., unpublished). They differ in the distribution assumed for the data (Poisson-like for PoissonSeq, negative binomial for edgeR, DESeq2 and EBSeq, and non-parametric for NOISeq) and the statistical test used by the method (Poisson goodness-of-fit for PoissonSeq, exact/likelihood ratio for edgeR, generalized linear model for DESeq2, empirical Bayesian for EBSeq and NOISeq). DEGs were obtained from a meta-analysis of the results from the previously five single selected methods. The Simes method was used, and genes with a combined *P*-value lower than 0.05 and a logFC (from edgeR method) higher than 1 or lower than -1 were considered as DEGs. Euler diagram was created in the VennDiagrams.net website^4^. Clustering and heatmap were performed by the ClustVis web tool^5^. Gene enrichment analyses were performed with the Fischer’s exact test using topGO package in R^6^ and the GO file retrieved from the PGSB/MIPS PlantsDB website.

### Comparative Analysis of DEGs

For Arabidopsis and tomato species, DEGs after mite attack were taken from Supplementary Material of corresponding publications (Zhurov et al., 2014; Martel et al., 2015). EnsemblPlants BioMart tool was used to obtain InterPro IDs and descriptions from the barley, Arabidopsis and tomato DEGs. Venn diagrams were created using the Venny 2.1 tool^7^. InterPro IDs network was constructed using Cytoscape v3.5.1^8^.

### Real-Time Reverse Transcription Quantitative PCR Analyses (RT-qPCR)

For real-time RT-qPCR studies, total RNA was isolated from barley leaves as described above. cDNAs were synthesized from 2 μg of RNA using the RevertAid H Minus First Strand cDNA Synthesis Kit (Thermo Scientific) following the manufacturer’s instructions. RT-qPCR analyses were performed for triplicated samples by means of a Light Cycler 480 real-time system (Roche) using the SYBR Green detection system (Roche). mRNA quantification was expressed as relative expression levels (2^-ΔCt^) (Livak and Schmittgen, 2001) and standardized to barley cyclophilin (*HvCycl* gene) mRNA levels (Diaz-Mendoza et al., 2014). Expression levels of the *T. urticae* Ribosomal Protein 49 (*TuRP49*) mRNA (Santamaria et al., 2015) were quantified as relative expression levels (2^-ΔCt^) by subtracting the ΔCt value of the barley cyclophilin from the ΔCt value of the mite probe to normalize for the amount of barley tissue present in each sample. To normalize for the amount of barley and mite tissue present in each sample, expression levels of the *T. urticae* Vitellogenin (*TuVTG*) and Autophagy related protein (*TuATG13*) were quantified as fold change levels (2^-ΔΔCt^) normalized to both, the barley cyclophilin and the *T. urticae* Ribosomal Protein 49. The primers used are shown in Supplementary Table 1. Efficiency of the primers was determined based on the slope of a standard curve and was between 97.5 and 102.5% for all primer pairs tested. Three plants from the three independent experiments were used per treatment.

### Statistical Analysis

Data related to physiological and biochemical characterization experiments were compared between control and stressed plants and analyzed by One-Way ANOVA followed by a many-to-one Dunnett’s *post hoc* test. In figures, one or three asterisks indicated significant differences (Dunnett’s test, *P* < 0.05 and *P* < 0.001, respectively). Expression analyses of mite performance were performed by One-Way ANOVA, followed by Student Newman–Keuls (SNK) *post hoc* test. In figures, different letters indicated significant differences (SNK test, *P* < 0.05).

## Results

### Combined Water Deprivation and Spider Mite Infestation Strengthen Alterations in the Plant Physiological Status

To establish a time point to further analyze the effects of dehydration and mites, 7-day-old barley plants were subjected to water deprivation, mite infestation or combined stresses for 4, 7, 10, and 13 days (Supplementary Figure 1). Phenotypic observations showed progressive deleterious effects caused by both treatments. Mite feeding produced chlorotic spots and induced yellowish of the apical part of the leaves. Dehydration induced a delay in plant growth and a progressive whiter of the plant, which was reinforced when combined with mites stress. As at 10 days of dehydration and mites combined stresses plants were too damaged to guarantee a complete molecular analysis, 7 days was selected as the time point to analyze the effects caused by mites and dehydration.

**FIGURE 1:**
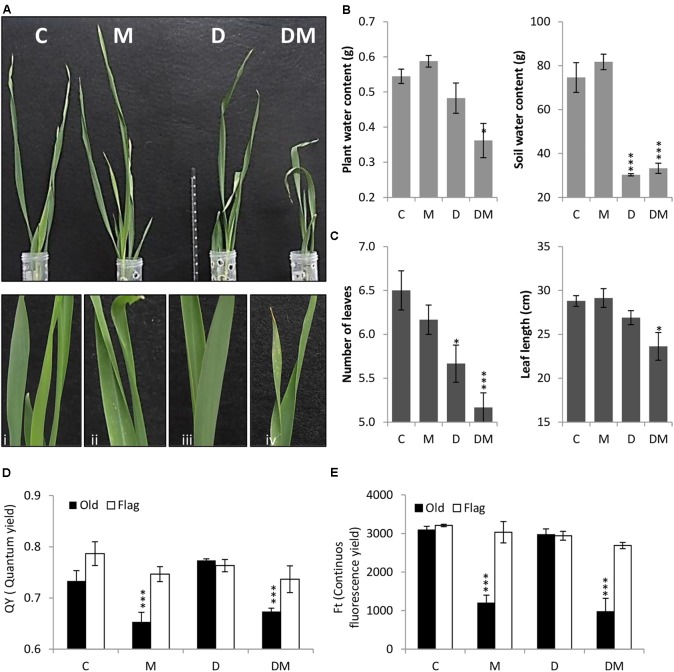
Phenotypical, physiological and biochemical parameters of barley plants 7 days post treatment. **(A)** Phenotype of barley plants 7 days post spider mite infestation (M), dehydration (D), spider mite infestation + dehydration (DM) or control conditions (C) after taking out plastic cylinders to avoid mite dispersion. Plant details are showed in i, ii, iii, and iv. **(B)** Plant/Soil water content (g). **(C)** Number of leaves and leaf length (cm). **(D)** Quantum yield of the photosystem II (QY). **(E)** Continuous fluorescence yield (FT). Data represent the mean ± SE of *n* = 3 replicates. Asterisks indicate significant differences among control plants and each treatment determined by a One-way ANOVA test (*P* < 0.05, Dunnett’s *post hoc* test).

After 7 days of treatment, plant phenotypes were analyzed and physiological and biochemical parameters studied. Plant images corroborated the deleterious effects of both stresses and detailed images highlight the chlorotic spots caused by spider mite infestation (**Figure 1A**). As expected, SWC was significantly lower in dehydration stressed samples (**Figure 1B**). Dehydration effects were potentiated by mite stress. Combined stresses lead to a higher reduction in PWC, leaf length and leaf number than that observed with only water deprivation (**Figures 1B,C**). When mites were included in the treatment, a significant reduction in the QY and in the continuous FT was observed in the oldest leaf (**Figures 1D,E**).

### Single or Multiple Stresses Differentially Affect the Expression of Barley Genes

To obtain more information on the changes in gene expression associated to the response of the barley plant to the different stresses, a RNA-seq analysis was performed in plants subjected to spider mite infestation (M), dehydration (D), mite infestation and dehydration (DM), or maintained under control conditions (C) during 7 days. In order to reduce the impact of intrinsic disparity between analytical methods and gene length bias, differential expressed genes (DEGs) were obtained in the RNA-seq 2G platform using a meta-analysis of the results from five selected methods (Supplementary Data Sheet 1).

**FIGURE 2:**
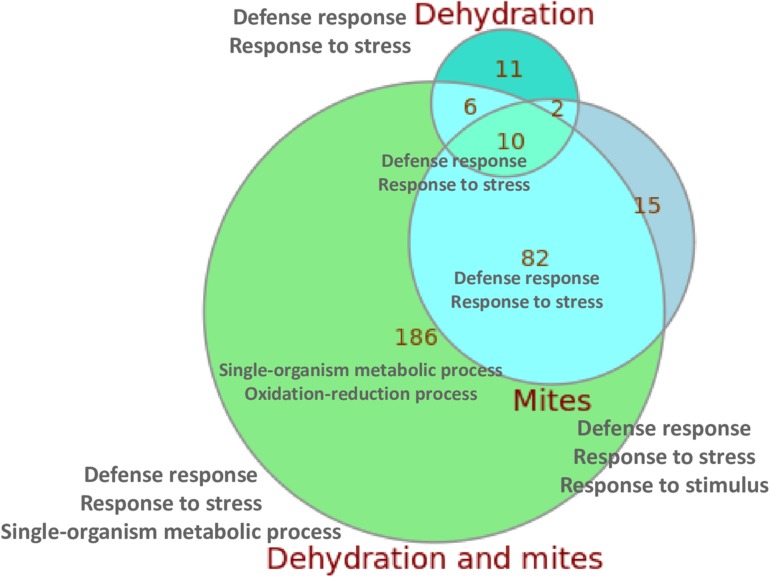
Euler diagram showing the number of specific and shared DEGs detected after the different treatments. The main enriched GO categories for each group/subgroup are indicated.

When compared with control plants, the highest number of DEGs was found in the plants subjected to simultaneous mite and dehydration treatments (284 genes), and the lowest in the dehydration stressed plants (29 genes). **Figure 2** summarizes the overlaps in the DEGs in response to the different treatments. From the 284 DEGs between C and DM conditions, 186 were not differentially expressed in response to mites or dehydration. On the contrary, 92 of the 109 DEGs in response to mites and 16 of the 29 DEGs in response to dehydration were shared by DM treatment (a complete list of DEGs for subgroups is compiled in Supplementary Data Sheet 2). Whereas most DEGs were up-regulated after mite (90.8%) or mite/dehydration (86.3%) treatments, a similar number of genes were up- or down-regulated after dehydration stress (55.2 and 44.8%) (Supplementary Figure 2). All shared DEGs were regulated in the same direction with the exception of a glucan endo-1,3-beta glucosidase and an undescribed protein that were down-regulated in the dehydration treatment and up-regulated in the M and DM treatments, respectively. Many up-regulated genes in both, the mites and dehydration and mites treated plants, were involved in the jasmonic acid pathway. Several genes are related to jasmonate biosynthesis (allene oxide synthase, allene oxide cyclase, lipoxygenases), and a higher number was related to jasmonate response (protease inhibitors, thionins, not specified jasmonate-induced proteins). Ten genes were differentially expressed after the three (M, D, and DM) treatments. All of these shared DEGs were up-regulated and included genes of the defense-related thionin family.

When we focused in the relationships of DEGs with specific biological processes using enrichment Gene Ontology (GO) analysis, several findings were observed (Supplementary Table 2). In all comparisons, with the exception of M/DM, an enrichment of the biological categories related to defense response, response to (biotic) stress or response to (biotic) stimulus were reported. When DEGs between M/DM treatments were compared, an enrichment of GO terms related with the response to abiotic (dehydration) stimuli was found. In addition, an enrichment of DEGs related to oxidation-reduction processes was present in the C/DM comparison. The two/three GO enriched terms with the lowest *p*-values for the control/treatment comparisons and for the overlapping subgroups are included in **Figure 2** (a complete list of enriched GO terms for subgroups is compiled in Supplementary Data Sheet 2).

**FIGURE 3:**
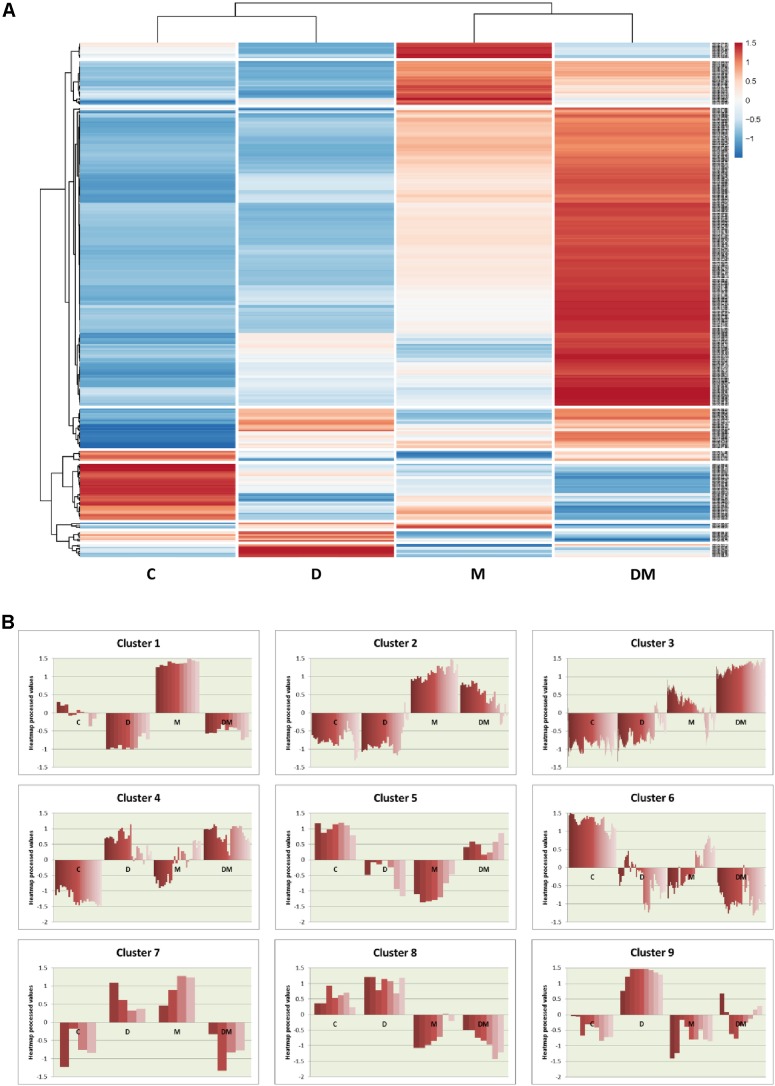
Hierarchical clustering of differentially expressed genes among the different treatments. **(A)** Heatmap showing normalized color intensity from the expression values of the 350 DEGs. **(B)** Main clusters identified from hierarchical gene tree cluster analysis showing heatmap expression processed values for every gene after the different treatments. C, control; D, dehydration; M, mites; DM, dehydration and mites.

To further analyze the expression of the DEGs, a heatmap was performed. This heatmap shows the relative expression values for the 350 DEGs obtained from at least one treatment comparison (**Figure 3A**). Globally, the heatmap highlights the greater changes in gene expression following mite infestation, which are reinforced by dehydration. Control and dehydration samples were grouped together, as well as mites and dehydration and mites samples. Clustering showed the presence of groups of genes similarly regulated (**Figure 3B** and Supplementary Data Sheet 3). A high number of genes are grouped in cluster 3. These genes are hugely induced by the DM combined stress. Whereas most of them are also induced by mite single stress (3.1 cluster), a small subset showed lower expression in mites and control than in dehydration treatment (3.2 cluster). Many GO terms related to defense and stress responses are enriched in this group. Cluster 1 includes genes induced by mites and repressed by dehydration, with enrichment in genes related to the response to oxidative stress. Cluster 2 is formed by genes more induced after mite stress than after DM stress and includes several genes related to carbohydrate metabolic processes. The rest of clusters did not present any enrichment GO term but present interesting features. For example, cluster 6 comprises genes repressed by any stress, and clusters 4 and 9 are formed by genes induced after dehydration and mites and dehydration treatments or only by water-deficit stress, respectively.

### Protein Domains From Differentially Expressed Genes in Response to Mites Are Quite Conserved in Barley, Arabidopsis and Tomato

**FIGURE 4:**
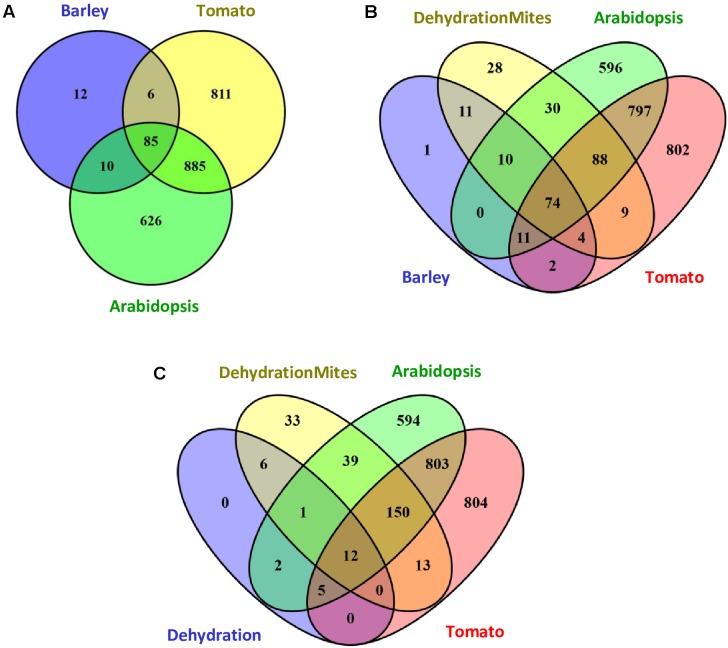
Venn diagrams showing the number of specific and shared Interpro identifiers. **(A)** Interpro identifiers from DEGs after mites treatment in Arabidopsis, tomato and barley. **(B)** Interpro identifiers from DEGs after mites treatment in Arabidopsis, tomato and barley, including the overlap with the dehydration and mites treatment in barley. **(C)** Interpro identifiers from DEGs after dehydration and dehydration and mites treatments in barley including the overlap with the mites treatment in Arabidopsis and tomato.

The response of barley to mite stress was compared with the response to mites previously reported for Arabidopsis and tomato (Zhurov et al., 2014; Martel et al., 2015). The functional domains present in the proteins encoded by DEGs were retrieved from the Interpro annotations in the EnsemblPlants database. The higher number of DEGs reported in Arabidopsis and tomato in response to *T. urticae* correlated with a higher number of Interpro annotated domains. Whereas 1606 and 1787 different Interpro identifiers were obtained from Arabidopsis and tomato, respectively, 113 were identified in barley. When Interpro domain identifiers were compared (**Figure 4A**), Arabidopsis and tomato only shared with barley the 60.4% and a 54.3% of identifiers, respectively. On the contrary, a 75.2% of the barley identifiers were shared by the three species, and an 89.4% was shared by barley and Arabidopsis or tomato. A schematic network highlighting the Interpro identifiers not shared by the three species is shown in Supplementary Figure 3. Supplementary Data sheet 4 includes the Interpro identifiers for each comparison involving barley data.

To obtain more information on the meaning of the 12 Interpro identifiers specific for barley, the functional domains present in the proteins encoded by DEGs after the DM combined treatment were added to the comparison (**Figure 4B**). Eleven of the 12 identifiers were also detected in proteins encoded by DEGs from the combined stresses, suggesting they are domains involved in the response of barley to mites. Further analysis of this comparison indicated that 226 of 254 Interpro identifiers from the DEGs after combined stresses were also identified after mite treatment of barley, Arabidopsis or tomato. The rest 28 identifiers were only recorded after combined treatment and could be involved in the response to dehydration stress.

To check this hypothesis, Interpro identifiers found in the proteins encoded by DEGs after dehydration treatment were compared to those coming from barley dehydration and mites treatment, and Arabidopsis and tomato mite infestation (**Figure 4C**). Twenty of the 26 barley identifiers were also detected after mite treatment of Arabidopsis or tomato. Three of the other six identifiers were common to barley D, M, and DM categories (domains corresponding to ribosomal proteins). Only three identifiers were specific from D and DM categories, which are present in adenylate kinase proteins, and could be assigned specifically to the dehydration stress response.

### Combined Biotic-Abiotic Stresses Influences the Time Course Response of Mites and Dehydration Induced Genes

**FIGURE 5:**
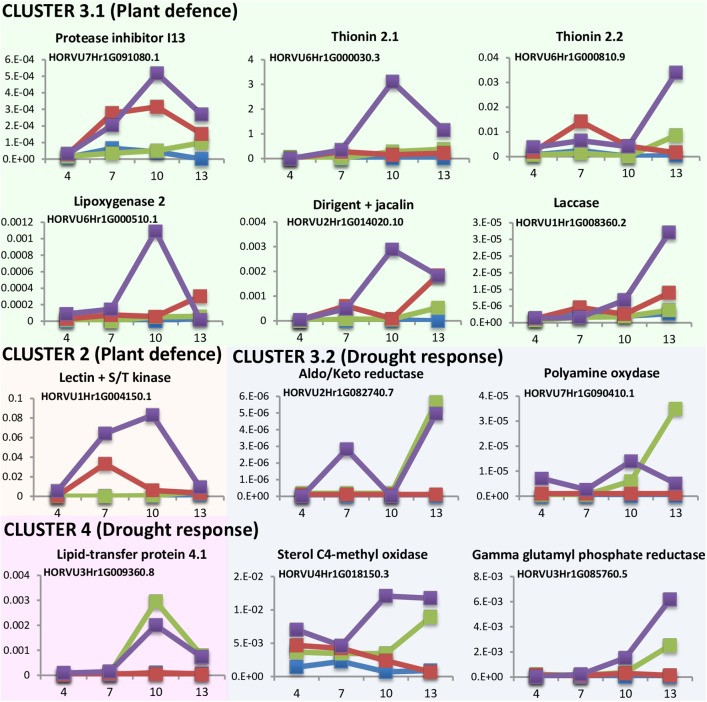
Messenger expression levels of selected differentially expressed barley genes after 4, 7, 10, and 13 days of stress treatment assayed by RT-qPCR. Total RNA was extracted from leaves after dehydration treatment (green), mites treatment (red), dehydration and mites treatment (purple) and non-treated leaves (blue). Data were expressed as relative mRNA levels normalized to barley *cyclophilin* mRNA content. Genes from the same cluster identified in the hierarchical analysis have the same background color.

DEGs analyses point to a quicker response to mite stress than that observed after dehydration stress. This response is reinforced in water deprivation conditions. To further characterize the time course of these responses the expression levels of several DEGs was quantified at different time points upon stress conditions (**Figure 5**). Twelve genes were selected in function of their expression patterns upon the stress treatments (Supplementary Figure 4). Most genes were from cluster 3, with the highest expression after the dehydration and mites treatment. Six of these genes were selected from the large subset of genes with a higher expression in mites than in dehydration or control categories (3.1 cluster), and correspond with genes from families largely known to be involved in plant defense (a protease inhibitor, a lipoxygenase and two thionins), a gene encoding a protein with a lectin (jacalin) domain and a dirigent domain, with homologs previously related to plant defense (Weidenbach et al., 2016), and a protein with a laccase domain, which is involved in lignification and resistance to pathogens and pests (Hu et al., 2017). Four genes belong to the small subset of genes with lower expression in mites and control than in dehydration categories (3.2 cluster) and correspond with genes encoding enzymes previously related to pathways involved in drought response in plants, such are aldo/keto reductases, polyamine oxydases, sterol C4-methyl oxidases, and gamma glutamyl phosphate reductases (Liang et al., 2013; Hatmi et al., 2015; Kumar et al., 2015; Sengupta et al., 2015). One gene was selected from cluster 2. This gene encodes a protein combining the known defensive carbohydrate-bound protein lectin domain with a serine/threonine kinase signaling domain and was previously related to plant defense (Bouwmeester et al., 2011). The last one (encoding a lipid transfer protein) belongs to cluster 4, formed by genes induced by dehydration and dehydration and mites treatments. This family of proteins has been associated to drought tolerance (Guo et al., 2013).

Results pointed out interesting features (**Figure 5**). All genes putatively induced by mites (clusters 2 and 3.1) were more expressed after the combined dehydration and mite treatments than after the single mite treatment. Besides, whereas their expression always peaks after 10 or 13 days of MD treatment, the expression of some genes peaks after 7 days of M treatment. The dehydration-related genes (clusters 3.2 and 4) showed peaks of expression at 10 or 13 days after both, D and DM treatments. However, most genes were induced earlier after combined MD treatment than after single dehydration treatment.

### Mite Population Development Depends on the Water Availability for the Plant

**FIGURE 6:**
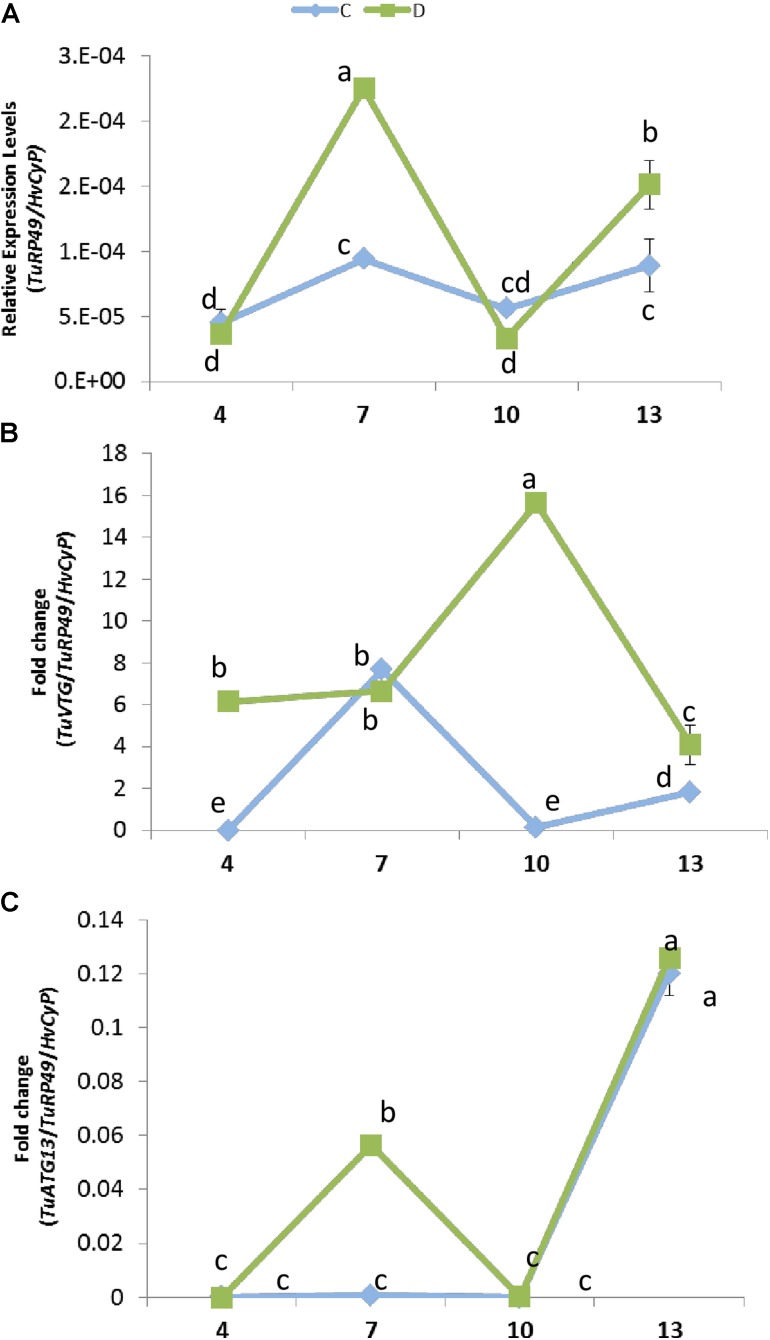
Effect of barley dehydration on *T. urticae* performance. **(A)** Quantification of *T. urticae* Ribosomal Protein 49 (*TuRP49*) mRNA expression. **(B)** *TuVTG* (Vitellogenin) mRNA expression normalized to the spider mite population (*TuRP49*). **(C)** *TuATG13* (Autophagy related protein) mRNA expression normalized to the spider mite population (*TuRP49*). Expression levels were quantified 4, 7, 10, and 13 days post infestation under control or dehydration stress conditions. Different letters indicate significant differences determined by a One-way ANOVA test (*P* < 0.05, Student Newman–Keuls *post hoc* test).

The expression of plant genes in response to mite infestation is affected by dehydration or well-watered conditions. To check its consequences on the mite population dynamics, the presence of the mite at different time points of infestation was analyzed by quantifying *T. urticae* Ribosomal Protein 49 (*TuRP49*) mRNA levels (**Figure 6A**). Total mite population increased from 4 to 7 days after infestation, growth that was stronger in water deprivation condition. Then, the mite population decreased markedly from 7 to 10 days in both conditions. Finally, mites increased again from 10 to 13 days, achieving a higher level in dehydration than in well-watered conditions.

To further analyze this timely up and down behavior, an approximation to the ratio of females/males was performed by quantifying the expression of a gene encoding a vitellogenin, which is mainly expressed in females (Cabrera et al., 2009) (**Figure 6B**). When plants were placed in dehydration conditions, the estimated amount of females was already high after 4 days of infestation and presented a marked peak at 10 days. On the contrary, when water was present, the estimated amount of females was scarce at 4 and 10 days, and peaks at 7 days of treatment. Likewise, the prevalence of egg stage was assessed by quantifying the expression of a gene encoding an autophagy related protein predominantly expressed in eggs (ORCAE *T. urticae* website^9^) (**Figure 6C**). Whereas in water deprivation the estimated amount of eggs showed two peaks, at 7 and 13 days of treatment, the amount of eggs was scarce in full water treatment and was only detected after 13 days of infestation.

## Discussion

Understanding how herbivores behave in field conditions is crucial to establish accurate integrated pest management actions. Since field conditions are complex and variable, analysis of small pieces of this natural scenario may help to understand the whole system. As drought and spider mites are two abiotic/biotic stresses predicted to increase crop losses under current climate change, the knowledge of plant responses to a combination of both stresses could help future crop production strategies.

The outcome of stress experiments depends on the relative severity and duration of the stresses applied, and the developmental stage of the plant (Zhang and Sonnewald, 2017). In our experimental conditions, water deprivation produces stronger phenotypical effects than mite stress at 10–13 days of treatment, with a remarkable reduction in plant size and an advanced withered state. However, at 7 days of treatment, mild phenotypical impact was observed. While dehydration provokes a delay in growth, with a lower number of leaves, mite feeding produced a decrease in photosynthesis efficiency. Water deficit consequences were exacerbated when combined stresses were applied. Thus, transcriptomic responses were expected for all stress combinations at this time point. Early responses are common across many different treatments. Changes in Ca_2_^+^ signatures, sugar signals, reactive oxygen species (ROS) and phytohormone levels are triggered by most biotic and abiotic stresses (Atkinson and Urwin, 2012; Sham et al., 2015; Nguyen et al., 2016b). However, depending on the environmental stimuli, specific subsets of genes respond. In order to guarantee survival and reproductive success, this fine tuning permits to prioritize resources toward growth or defense when plants are subjected to different stresses (Denancé et al., 2013; Huot et al., 2014).

Intriguingly, mite attack induced stronger transcriptomic changes than water deprivation. Whereas the expression of 109 genes was modified by mite treatment, only 29 responded to mild dehydration. These data suggest that changes in the expression of a reduced number of genes are enough to retard plant growth, but a broader remodeling of gene expression is needed to establish appropriate defensive programs against mites. Since interactions between abiotic and biotic stress occur at multiple levels in plants, it is difficult to predict overlaps between signaling pathways induced by single and combined stresses. In fact, molecular changes triggered by the application of simultaneous or sequential stresses differ from those induced by the individual stress (Atkinson et al., 2013; Prasch and Sonnewald, 2013; Rasmussen et al., 2013; Suzuki et al., 2014; Coolen et al., 2016; Davila Olivas et al., 2016). Furthermore, the number of regulated features usually increased with the complexity of the stresses applied. For example, the number of regulated features in individual drought and virus stress in Arabidopsis plants was 518 and 682, respectively, but rose to 1,744 when both stresses were applied in combination (Prasch and Sonnewald, 2013).

In our experimental conditions, the number of DEGs reached 284 when both dehydration and mites were applied. Jasmonic acid is the main hormone involved in the establishment of spider mite-induced defense responses in plant species (Ament et al., 2004; Zhurov et al., 2014; Martel et al., 2015; Santamaría et al., 2017). As expected, many up-regulated genes in both, the mites and dehydration and mites treated plants, were involved in the jasmonic acid pathway. At least, a half of the fifty most up-regulated genes were associated to jasmonic acid response. Many of them encode defensive proteins such as protease inhibitors and thionins with putative deterrent effects on herbivores. The actual role of some of these genes in the defensive mechanism triggered by jasmonic acid is also supported by their up-regulation in lines over-expressing the lipoxygenase involved in jasmonate biosynthesis (Losvik et al., 2017). Furthermore, the DEGs specific for the dehydration and mites treatment were enriched in functional categories related to both, biotic and abiotic responses. These data suggest an extensive remodeling of metabolic processes when both stresses are combined. This synergistic response overtakes gene expression adjustment triggered by individual stresses. Besides, many DEGs suffered a higher induction of their expression when mite infestation was done in dehydration conditions than in well-watered plants. This feature was previously described (Nguyen et al., 2016a). The expression of DEGs was higher in *Solanum dulcamara* plants infested with the caterpillar *Spodoptera exigua* in drought conditions. Authors pointed out a role for secondary defensive metabolites in controlling other physiological processes. In our dataset, the defensive molecules thionins were also up-regulated after water deprivation. If thionins could have a role in dehydration tolerance or their induction by water deprivation is related to reinforce cell walls against possible pathogen/pest attacks remains to be elucidated. The temporal expression patterns of several genes induced by mites confirmed the stronger gene induction caused by the synergistic effect of mites and dehydration, which roughly starts at the same time than that following individual mite stress, but is maintained at higher levels during more time. On the contrary, dehydration gene regulation seems to start earlier in the combined treatment but reaches similar expression levels than that triggered by the individual dehydration stress.

As the synergistic behavior of dehydration and mites stresses leads to a broader and higher differential expression than that caused by individual stresses, the assignment of specific DEGs to dehydration or mite response was not possible by comparing individual and combined transcriptomic results. As an alternative, previous characterization of tomato and Arabidopsis responses to *T. urticae* were explored (Zhurov et al., 2014; Martel et al., 2015). Interpro identifiers were used to establish if common domains were carried out by the induced/repressed proteins after mites’ infestation, and then, to discover dehydration-related domains by comparisons using the DEGs after dehydration and dehydration and mites treatments. Although profound divergences were found in the spider mite-induced responses between tomato and Arabidopsis (Martel et al., 2015), most barley domains present in proteins regulated by mite infestation were shared with those reported from tomato and Arabidopsis. The only barley-specific domain previously related to plant defense was the Bowman–Birk protease inhibitory domain, which was absent in the proteins encoded by the Arabidopsis and tomato genomes, MEROPS database (Rawlings et al., 2016). When dehydration and dehydration and mites results were added, a set of 28 Interpro identifiers were not shared with those from barley, Arabidopsis or tomato mite-treated plants. Six of them were regulated after both, dehydration and dehydration and mites treatments. This set includes the adenylate kinase domain, previously related to drought response in tomato (Gong et al., 2010). From the other 22 Interpro identifiers, only found after the dehydration and mites treatment, the cystatin domain from cysteine protease inhibitor proteins was reported to be differentially expressed in response to both, water deprivation and herbivore attack (Diaz-Mendoza et al., 2016; Martinez et al., 2016); the glutathione synthase domain is involved in the production of glutathione, which is an anti-oxidant protective compound with a critical role in plant resistance to biotic and abiotic stresses (Noctor et al., 2012); and the gamma-glutamyl phosphate reductase domain catalyzes the second step in the biosynthesis of the amino acid proline from glutamate. Proline is an osmoprotectant and signaling molecule largely accumulated in response to abiotic stresses, but also with a role in the response to biotic stresses (Szabados and Savouré, 2010). These ambiguities remark the difficulties to assign the Interpro identifiers from the DEGs after combined stresses specifically to the dehydration or the mite response.

**FIGURE 7:**
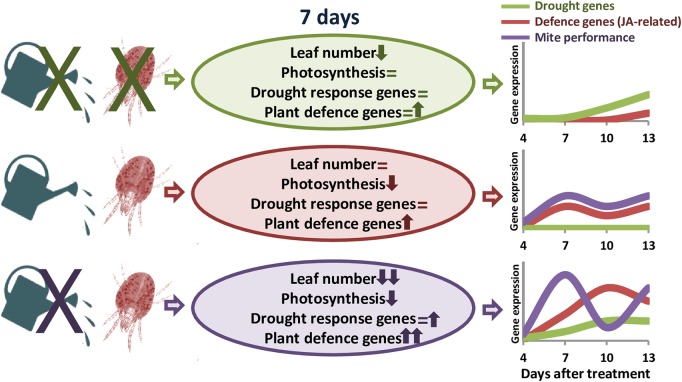
Schematic model summarizing the main effects of dehydration and spider mite treatments. Ovals include information on the variations found in plant phenotype features and gene expression. Up arrows, down arrows and equal signs mean positive effect, negative effect or no-effect, respectively. Line drawings show the predicted time course of the expression of defense and drought-related genes, and the performance of the mite throughout the different treatments.

Transcriptomic analyses in barley revealed a stronger defensive response to the aphid *Myzus persicae* than to the aphid *Rhopalosiphum padi*, which was correlated to the fact that barley is a good-host of *R. padi* but a poor-host of *M. persicae* (Escudero-Martinez et al., 2017). Likewise, *S. exigua* performed less well on *S. dulcamara* drought-stressed plants than on well-watered plants, which was correlated to stronger induction of plant herbivore-induced processes in drought conditions (Nguyen et al., 2016a). Thus, we will expect a worse performance of *T. urticae* in dehydration-stressed plants, since the defensive response of the plant is considerably higher than in well-watered conditions. However, mite performance was higher in water-stressed barley plants, as was previously reported for mites feeding on drought-stressed tomato plants (Ximénez-Embún et al., 2016, 2017a,b). Different hypothesis have been postulated to establish the consequences of drought stress on herbivore performance. In particular, the “plant stress hypothesis” states that drought causes the plant to have a higher nutritional value for herbivores, and the “plant vigor hypothesis” associates a reduction in growth and an increase in defense compounds caused by drought to a lower suitability for herbivores’ feeding (White, 2009). In maize, simultaneous soil drought and *T. urticae* infestation elevates the amount of proteins that enable maize to maintain the efficiency of photosynthesis and metabolism, as well as to protect its cells against metabolic injuries (Dworak et al., 2016). Besides, drought increases the improved nutritional value of tomato leaves by accumulating free amino acid and sugars, and altering hormonal balance (Ximénez-Embún et al., 2016, 2017b). In transgenic barley plants overexpressing the cysteine protease HvPap-1, a higher susceptibility to *T. urticae* correlated with a higher induction of protease inhibitors (Diaz-Mendoza et al., 2017). These findings suggest a mixed scenario where mite performance depends on an optimal balance of nutrients in the plant and its adaptation to plant defense compounds (Gutbrodt et al., 2011). In barley, drought effects on nutrients availability could be stimulating mite feeding, which would provoke a strong induction of plant defenses. However, the accumulation of proteins potentially required to resist the biotic attack would not be enough to increase plant resistance. Population dynamics supports this hypothesis, as total mite population showed temporal variations caused by the developmental cycle. In *T. urticae*, local mate competition conditioned sex-allocation strategies and favors female-biased sex ratios (Macke et al., 2011). Under dehydration conditions, higher mite populations are correlated to an elevated ratio of eggs, which are mostly fertilized to produce females to favor a quick increment of the population to take advantage of higher nutrient availability. In well-watered conditions, peaks of females and eggs ratios are temporally more distant, suggesting a larger developmental cycle and a slower increment of the mite population. **Figure 7** summarizes the main findings of this work. These findings highlight the striking differential behavior of both, the barley plant and the spider mite, depending on the watered state of the plant.

## Conclusion

Results demonstrate the enhanced performance of spider mite in barley under water deficit conditions. Unexpectedly, this behavior was concomitant to an apparent higher up-regulation of mite-induced defenses, which expression patterns were earlier modified than that related to dehydration tolerance signaling pathways. Thus, a stronger transcriptomic effort to manage an herbivore-stress should not be directly correlated to a higher plant resistance. Complex signaling networks leading to metabolic alterations affected by combined stresses should be in the focus to really understand and predict the consequences of actual stresses in crop production.

## Author Contributions

ID and MM conceived the research. MS performed most of the experimental research. MS, ID, and MM participated in the design, acquisition, analysis, and interpretation of data for the work. All authors contributed to the final version of the manuscript.

## Conflict of Interest Statement

The authors declare that the research was conducted in the absence of any commercial or financial relationships that could be construed as a potential conflict of interest.

## Footnotes

1 https://www.ebi.ac.uk/arrayexpress/

2 http://pgsb.helmholtz-muenchen.de/plant/barley/index.jsp

3 http://52.90.192.24:3838/rnaseq2g/

4 http://www.venndiagrams.net/

5 http://biit.cs.ut.ee/clustvis/

6 http://bioconductor.org/packages/release/bioc/html/topGO.html

7 http://bioinfogp.cnb.csic.es/tools/venny/

8 http://www.cytoscape.org/

9 http://bioinformatics.psb.ugent.be/orcae/overview/Tetur

## References

[B1] Agut B. Gamir J. Jaques J. A. Flors V. (2015). *Tetranychus urticae*-triggered responses promote genotype-dependent conspecific repellence or attractiveness in citrus. *New Phytol.* 207 790–804. 10.1111/nph.13357 25771705

[B2] Ament K. Kant M. R. Sabelis M. W. Haring M. A. Schuurink R. C. (2004). Jasmonic acid is a key regulator of spider mite-induced volatile terpenoid and methyl salicylate emission in tomato. *Plant Physiol.* 135 2025–2037. 10.1104/pp.104.048694 15310835 PMC520773

[B3] Atkinson N. J. Lilley C. J. Urwin P. E. (2013). Identification of genes involved in the response of Arabidopsis to simultaneous biotic and abiotic stresses. *Plant Physiol.* 162 2028–2041. 10.1104/pp.113.222372 23800991 PMC3729780

[B4] Atkinson N. J. Urwin P. E. (2012). The interaction of plant biotic and abiotic stresses: from genes to the field. *J. Exp. Bot.* 63 3523–3543. 10.1093/jxb/ers100 22467407

[B5] Barnabás B. Jäger K. Fehér A. (2008). The effect of drought and heat stress on reproductive processes in cereals. *Plant Cell Environ.* 31 11–38. 10.1111/j.1365-3040.2007.01727.x 17971069

[B6] Bensoussan N. Santamaria M. E. Zhurov V. Diaz I. Grbić M. Grbić V. (2016). Plant-herbivore interaction: dissection of the cellular pattern of *Tetranychus urticae* feeding on the host plant. *Front. Plant Sci.* 7:1105. 10.3389/fpls.2016.01105 27512397 PMC4961969

[B7] Bouwmeester K. de Sain M. Weide R. Gouget A. Klamer S. Canut H. (2011). The lectin receptor kinase LecRK-I.9 is a novel *Phytophthora* resistance component and a potential host target for a RXLR effector. *PLoS Pathog.* 7:e1001327. 10.1371/journal.ppat.1001327 21483488 PMC3068997

[B8] Bray N. L. Pimentel H. Melsted P. Pachter L. (2016). Near-optimal probabilistic RNA-seq quantification. *Nat. Biotechnol.* 34 525–527. 10.1038/nbt.3519 27043002

[B9] Cabrera A. R. Donohue K. V. Roe R. M. (2009). Regulation of female reproduction in mites: a unifying model for the Acari. *J. Insect Physiol.* 55 1079–1090. 10.1016/j.jinsphys.2009.08.007 19698719

[B10] Cantalapiedra C. P. García-Pereira M. J. Gracia M. P. Igartua E. Casas A. M. Contreras-Moreira B. (2017). Large differences in gene expression responses to drought and heat stress between elite barley cultivar Scarlett and a spanish landrace. *Front. Plant Sci.* 8:647. 10.3389/fpls.2017.00647 28507554 PMC5410667

[B11] Coolen S. Proietti S. Hickman R. Davila Olivas N. H. Huang P. P. Van Verk M. C. (2016). Transcriptome dynamics of Arabidopsis during sequential biotic and abiotic stresses. *Plant J.* 86 249–267. 10.1111/tpj.13167 26991768

[B12] Daryanto S. Wang L. Jacinthe P. A. (2016). Global synthesis of drought effects on maize and wheat production. *PLoS One* 11:e0156362. 10.1371/journal.pone.0156362 27223810 PMC4880198

[B13] Davila Olivas N. H. Coolen S. Huang P. Severing E. van Verk M. C. Hickman R. (2016). Effect of prior drought and pathogen stress on Arabidopsis transcriptome changes to caterpillar herbivory. *New Phytol.* 210 1344–1356. 10.1111/nph.13847 26847575

[B14] Dawson I. K. Russell J. Powell W. Steffenson B. Thomas W. T. Waugh R. (2015). Barley: a translational model for adaptation to climate change. *New Phytol.* 206 913–931. 10.1111/nph.13266 25605349

[B15] Delp G. Gradin T. Ahman I. Jonsson L. M. (2009). Microarray analysis of the interaction between the aphid *Rhopalosiphum padi* and host plants reveals both differences and similarities between susceptible and partially resistant barley lines. *Mol. Genet. Genomics* 281 233–248. 10.1007/s00438-008-0409-3 19085010

[B16] DeLucia E. H. Nabity P. D. Zavala J. A. Berenbaum M. R. (2012). Climate change: resetting plant-insect interactions. *Plant Physiol.* 160 1677–1685. 10.1104/pp.112.204750 22972704 PMC3510101

[B17] Denancé N. Sánchez-Vallet A. Goffner D. Molina A. (2013). Disease resistance or growth: the role of plant hormones in balancing immune responses and fitness costs. *Front. Plant Sci.* 4:155. 10.3389/fpls.2013.00155 23745126 PMC3662895

[B18] Diaz-Mendoza M. Velasco-Arroyo B. Gonzalez-Melendi P. Martinez M. Diaz I. (2014). C1A cysteine protease-cystatin interactions in leaf senescence. *J. Exp. Bot.* 65 3825–3833. 10.1093/jxb/eru043 24600023

[B19] Diaz-Mendoza M. Velasco-Arroyo B. Santamaria M. E. Diaz I. Martinez M. (2017). HvPap-1 C1A protease participates differentially in the barley response to a pathogen and an herbivore. *Front. Plant Sci.* 8:1585. 10.3389/fpls.2017.01585 28955371 PMC5601043

[B20] Diaz-Mendoza M. Velasco-Arroyo B. Santamaria M. E. González-Melendi P. Martinez M. Diaz I. (2016). Plant senescence and proteolysis: two processes with one destiny. *Genet. Mol. Biol.* 39 329–338. 10.1590/1678-4685-GMB-2016-0015 27505308 PMC5004835

[B21] Díaz-Riquelme J. Zhurov V. Rioja C. Pérez-Moreno I. Torres-Pérez R. Grimplet J. (2016). Comparative genome-wide transcriptome analysis of *Vitis vinifera* responses to adapted and non-adapted strains of two-spotted spider mite, *Tetranychus urticae*. *BMC Genomics* 17:74. 10.1186/s12864-016-2401-3 26801623 PMC4724079

[B22] Dworak A. Nykiel M. Walczak B. Miazek A. Szworst-Łupina D. Zagdańska B. (2016). Maize proteomic responses to separate or overlapping soil drought and two-spotted spider mite stresses. *Planta* 244 939–960. 10.1007/s00425-016-2559-6 27334025 PMC5018026

[B23] Escudero-Martinez C. M. Morris J. A. Hedley P. E. Bos J. I. B. (2017). Barley transcriptome analyses upon interaction with different aphid species identify thionins contributing to resistance. *Plant Cell Environ.* 40 2628–2643. 10.1111/pce.12979 28452058 PMC6084319

[B24] Fahad S. Bajwa A. A. Nazir U. Anjum S. A. Farooq A. Zohaib A. (2017). Crop production under drought and heat stress: plant responses and management options. *Front. Plant Sci.* 8:1147. 10.3389/fpls.2017.01147 28706531 PMC5489704

[B25] Farooq M. Wahid A. Kobayashi N. Fujita D. Basra S. M. A. (2009). Plant drought stress: effects, mechanisms and management. *Agron. Sustain Dev.* 29 185–212. 10.1051/agro:2008021

[B26] Foyer C. H. Rasool B. Davey J. W. Hancock R. D. (2016). Cross-tolerance to biotic and abiotic stresses in plants: a focus on resistance to aphid infestation. *J. Exp. Bot.* 67 2025–2037. 10.1093/jxb/erw079 26936830

[B27] Golldack D. Lüking I. Yang O. (2011). Plant tolerance to drought and salinity: stress regulating transcription factors and their functional significance in the cellular transcriptional network. *Plant Cell Rep.* 30 1383–1391. 10.1007/s00299-011-1068-0 21476089

[B28] Gong P. Zhang J. Li H. Yang C. Zhang C. Zhang X. (2010). Transcriptional profiles of drought-responsive genes in modulating transcription signal transduction, and biochemical pathways in tomato. *J. Exp. Bot.* 61 3563–3575. 10.1093/jxb/erq167 20643807 PMC2921197

[B29] Guo L. Yang H. Zhang X. Yang S. (2013). Lipid transfer protein 3 as a target of MYB96 mediates freezing and drought stress in Arabidopsis. *J. Exp. Bot.* 64 1755–1767. 10.1093/jxb/ert040 23404903 PMC3617838

[B30] Guo P. Baum M. Grando S. Ceccarelli S. Bai G. Li R. (2009). Differentially expressed genes between drought-tolerant and drought-sensitive barley genotypes in response to drought stress during the reproductive stage. *J. Exp. Bot.* 60 3531–3544. 10.1093/jxb/erp194 19561048 PMC2724701

[B31] Gutbrodt B. Mody K. Dorn S. (2011). Drought changes plant chemistry and causes contrasting responses in lepidopteran herbivores. *Oikos* 120 1732–1740. 10.1111/j.1600-0706.2011.19558.x

[B32] Hatmi S. Gruau C. Trotel-Aziz P. Villaume S. Rabenoelina F. Baillieul F. (2015). Drought stress tolerance in grapevine involves activation of polyamine oxidation contributing to improved immune response and low susceptibility to *Botrytis cinerea*. *J. Exp. Bot.* 66 775–787. 10.1093/jxb/eru436 25385768

[B33] Hu Q. Min L. Yang X. Jin S. Zhang L. Li Y. (2017). Laccase GhLac1 modulates broad-spectrum biotic stress tolerance via DAMP-triggered immunity. *Plant Physiol.* 176 1808–1823. 10.1104/pp.17.01628 29229698 PMC5813555

[B34] Huberty A. F. Denno R. F. (2004). Plant water stress and its consequences for herbivorous insects: a new synthesis. *Ecology* 85 1383–1398. 10.1890/03-0352

[B35] Huot B. Yao J. Montgomery B. L. He S. Y. (2014). Growth-defense tradeoffs in plants: a balancing act to optimize fitness. *Mol. Plant* 7 1267–1287. 10.1093/mp/ssu049 24777989 PMC4168297

[B36] Koricheva J. Larsson S. Haukioja E. (1998). Insect performance on experimentally stressed woody plants: a meta-analysis. *Annu. Rev. Entomol.* 43 195–216. 10.1146/annurev.ento.43.1.195 15012389

[B37] Kumar M. S. Ali K. Dahuja A. Tyagi A. (2015). Role of phytosterols in drought stress tolerance in rice. *Plant Physiol. Biochem.* 96 83–89. 10.1016/j.plaphy.2015.07.014 26233709

[B38] Liang X. Zhang L. Natarajan S. K. Becker D. F. (2013). Proline mechanisms of stress survival. *Antioxid. Redox Signal.* 19 998–1011. 10.1089/ars.2012.5074 23581681 PMC3763223

[B39] Livak K. J. Schmittgen T. D. (2001). Analysis of relative gene expression data using real-time quantitative PCR and the 2^-ΔΔ*C*_T_^ Method. *Methods* 25 402–408. 10.1006/meth.2001.1262 11846609

[B40] Lobell D. B. Schlenker W. Costa-Roberts J. (2011). Climate trends and global crop production since 1980. *Science* 333 616–620. 10.1126/science.1204531 21551030

[B41] Losvik A. Beste L. Glinwood R. Ivarson E. Stephens J. Zhu L. H. (2017). Overexpression and down-regulation of barley lipoxygenase *LOX2.2* affects jasmonate-regulated genes and aphid fecundity. *Int. J. Mol. Sci.* 18:E2765. 10.3390/ijms18122765 29257097 PMC5751364

[B42] Macke E. Magalhães S. Bach F. Olivieri I. (2011). Experimental evolution of reduced sex ratio adjustment under local mate competition. *Science* 334 1127–1129. 10.1126/science.1212177 22052976

[B43] Martel C. Zhurov V. Navarro M. Martinez M. Cazaux M. Auger P. (2015). Tomato whole genome transcriptional response to *Tetranychus urticae* identifies divergence of spider mite-induced responses between tomato and Arabidopsis. *Mol. Plant Microbe Interact.* 28 343–361. 10.1094/MPMI-09-14-0291-FI 25679539

[B44] Martinez M. Santamaria M. E. Diaz-Mendoza M. Arnaiz A. Carrillo L. Ortego F. (2016). Phytocystatins: defense proteins against phytophagous insects and acari. *Int. J. Mol. Sci.* 17:E1747. 10.3390/ijms17101747 27775606 PMC5085774

[B45] Maserti B. E. Del Carratore R. Croce C. M. Podda A. Migheli Q. Froelicher Y. (2011). Comparative analysis of proteome changes induced by the two spotted spider mite *Tetranychus urticae* and methyl jasmonate in citrus leaves. *J. Plant Physiol.* 168 392–402. 10.1016/j.jplph.2010.07.026 20926159

[B46] Mayer K. F. Waugh R. Brown J. W. Schulman A. Langridge P. Platzer M. (2012). A physical, genetic and functional sequence assembly of the barley genome. *Nature* 491 711–716. 10.1038/nature11543 23075845

[B47] Migeon A., Dorkeld F. (2006–2017). *Spider Mites Web: A Comprehensive Database for the Tetranychidae*. Available at: http://www.montpellier.inra.fr/CBGP/spmweb

[B48] Nakashima K. Yamaguchi-Shinozaki K. Shinozaki K. (2014). The transcriptional regulatory network in the drought response and its crosstalk in abiotic stress responses including drought, cold, and heat. *Front. Plant Sci.* 5:170. 10.3389/fpls.2014.00170 24904597 PMC4032904

[B49] Nguyen D. D’Agostino N. Tytgat T. O. Sun P. Lortzing T. Visser E. J. (2016a). Drought and flooding have distinct effects on herbivore-induced responses and resistance in *Solanum dulcamara*. *Plant Cell Environ.* 39 1485–1499. 10.1111/pce.12708 26759219

[B50] Nguyen D. Rieu I. Mariani C. van Dam N. M. (2016b). How plants handle multiple stresses: hormonal interactions underlying responses to abiotic stress and insect herbivory. *Plant Mol. Biol.* 91 727–740. 10.1007/s11103-016-0481-8 27095445 PMC4932144

[B51] Noctor G. Mhamdi A. Chaouch S. Han Y. Neukermans J. Marquez-Garcia B. (2012). Glutathione in plants: an integrated overview. *Plant Cell Environ.* 35 454–484. 10.1111/j.1365-3040.2011.02400.x 21777251

[B52] Nussbaumer T. Martis M. M. Roessner S. K. Pfeifer M. Bader K. C. Sharma S. (2013). MIPS PlantsDB: a database framework for comparative plant genome research. *Nucleic Acids Res.* 41 D1144–D1151. 10.1093/nar/gks1153 23203886 PMC3531202

[B53] Oñate-Sánchez L. Vicente-Carbajosa J. (2008). DNA-free RNA isolation protocols for Arabidopsis thaliana, including seeds and siliques. *BMC Res. Notes* 1:93. 10.1186/1756-0500-1-93 18937828 PMC2613888

[B54] Praba M. L. Cairns J. E. Babu R. C. Lafitte H. R. (2009). Identification of physiological traits underlying cultivar differences in drought tolerance in rice and wheat. *J. Agron. Crop Sci.* 195 30–46. 10.1111/j.1439-037X.2008.00341.x

[B55] Prasch C. M. Sonnewald U. (2013). Simultaneous application of heat, drought, and virus to Arabidopsis plants reveals significant shifts in signaling networks. *Plant Physiol.* 162 1849–1866. 10.1104/pp.113.221044 23753177 PMC3729766

[B56] Ramegowda V. Senthil-Kumar M. (2015). The interactive effects of simultaneous biotic and abiotic stresses on plants: mechanistic understanding from drought and pathogen combination. *J. Plant Physiol.* 176 47–54. 10.1016/j.jplph.2014.11.008 25546584

[B57] Rasmussen S. Barah P. Suarez-Rodriguez M. C. Bressendorff S. Friis P. Costantino P. (2013). Transcriptome responses to combinations of stresses in Arabidopsis. *Plant Physiol.* 161 1783–1794. 10.1104/pp.112.210773 23447525 PMC3613455

[B58] Rawlings N. D. Barrett A. J. Finn R. (2016). Twenty years of the MEROPS database of proteolytic enzymes, their substrates and inhibitors. *Nucleic Acids Res.* 44 D343–D350. 10.1093/nar/gkv1118 26527717 PMC4702814

[B59] Santamaria M. E. Gonzalez-Cabrera J. Martinez M. Grbic V. Castanera P. Diaz I. (2015). Digestive proteases in bodies and faeces of the two-spotted spider mite, *Tetranychus urticae*. *J. Insect Physiol.* 78 69–77. 10.1016/j.jinsphys.2015.05.002 25960286

[B60] Santamaría M. E. Martinez M. Arnaiz A. Ortego F. Grbic V. Diaz I. (2017). MATI, a novel protein involved in the regulation of herbivore-associated signaling pathways. *Front. Plant Sci.* 8:975. 10.3389/fpls.2017.00975 28649257 PMC5466143

[B61] Sengupta D. Naik D. Reddy A. R. (2015). Plant aldo-keto reductases (AKRs) as multi-tasking soldiers involved in diverse plant metabolic processes and stress defense: a structure-function update. *J. Plant Physiol.* 179 40–55. 10.1016/j.jplph.2015.03.004 25840343

[B62] Sham A. Moustafa K. Al-Ameri S. Al-Azzawi A. Iratni R. AbuQamar S. (2015). Identification of Arabidopsis candidate genes in response to biotic and abiotic stresses using comparative microarrays. *PLoS One* 10:e0125666. 10.1371/journal.pone.0125666 25933420 PMC4416716

[B63] Suzuki N. Rivero R. M. Shulaev V. Blumwald E. Mittler R. (2014). Abiotic and biotic stress combinations. *New Phytol.* 203 32–43. 10.1111/nph.12797 24720847

[B64] Svoboda P. Janská A. Spiwok V. Prášil I. T. Kosová K. Vítámvás P. (2016). Global scale transcriptional profiling of two contrasting barley genotypes exposed to moderate drought conditions: contribution of leaves and crowns to water shortage coping strategies. *Front. Plant Sci.* 7:1958. 10.3389/fpls.2016.01958 28083001 PMC5187378

[B65] Szabados L. Savouré A. (2010). Proline: a multifunctional amino acid. *Trends Plant Sci.* 15 89–97. 10.1016/j.tplants.2009.11.009 20036181

[B66] Tariq M. Wright D. J. Bruce T. J. Staley J. T. (2013). Drought and root herbivory interact to alter the response of above-ground parasitoids to aphid infested plants and associated plant volatile signals. *PLoS One* 8:e69013. 10.1371/journal.pone.0069013 23894394 PMC3716814

[B67] Van Leeuwen T. Dermauw W. (2016). The molecular evolution of xenobiotic metabolism and resistance in chelicerate mites. *Annu. Rev. Entomol.* 61 475–498. 10.1146/annurev-ento-010715-023907 26982444

[B68] Wehner G. Balko C. Humbeck K. Zyprian E. Ordon F. (2016). Expression profiling of genes involved in drought stress and leaf senescence in juvenile barley. *BMC Plant Biol.* 16:3. 10.1186/s12870-015-0701-4 26733420 PMC4702385

[B69] Weidenbach D. Esch L. Möller C. Hensel G. Kumlehn J. Höfle C. (2016). Polarized defense against fungal pathogens is mediated by the jacalin-related lectin domain of modular poaceae-specific proteins. *Mol. Plant* 9 514–527. 10.1016/j.molp.2015.12.009 26708413

[B70] White T. C. R. (2009). Plant vigour versus plant stress: a false dichotomy. *Oikos* 118 807–808. 10.1111/j.1600-0706.2009.17495.x

[B71] Ximénez-Embún M. G. Castañera P. Ortego F. (2017a). Drought stress in tomato increases the performance of adapted and non-adapted strains of *Tetranychus urticae*. *J. Insect Physiol.* 96 73–81. 10.1016/j.jinsphys.2016.10.015 27789296

[B72] Ximénez-Embún M. G. Glas J. J. Ortego F. Alba J. M. Castañera P. Kant M. R. (2017b). Drought stress promotes the colonization success of a herbivorous mite that manipulates plant defenses. *Exp. Appl. Acarol.* 73 297–315. 10.1007/s10493-017-0200-4 29188401 PMC5727147

[B73] Ximénez-Embún M. G. Ortego F. Castañera P. (2016). Drought-stressed tomato plants trigger bottom-up effects on the invasive Tetranychus evansi. *PLoS One* 11:e0145275. 10.1371/journal.pone.0145275 26735490 PMC4703393

[B74] Zeng X. Bai L. Wei Z. Yuan H. Wang Y. Xu Q. (2016). Transcriptome analysis revealed the drought-responsive genes in Tibetan hulless barley. *BMC Genomics* 17:386. 10.1186/s12864-016-2685-3 27207260 PMC4875595

[B75] Zhang H. Sonnewald U. (2017). Differences and commonalities of plant responses to single and combined stresses. *Plant J.* 90 839–855. 10.1111/tpj.13557 28370754

[B76] Zhurov V. Navarro M. Bruinsma K. A. Arbona V. Santamaria M. E. Cazaux M. (2014). Reciprocal responses in the interaction between Arabidopsis and the cell-content-feeding chelicerate herbivore spider mite. *Plant Physiol.* 164 384–399. 10.1104/pp.113.231555 24285850 PMC3875816

