# Supplementary material for: Dehydration Stress Contributes to the Enhancement of Plant Defense Response and Mite Performance on Barley
Source: Front Plant Sci. 2018 Apr 6;9:458. doi: 10.3389/fpls.2018.00458 (PMC5898276; doi:10.3389/fpls.2018.00458)
Supplement: Supplementary file 1 [file Presentation_1.pdf]

Supplementary Material

Dehydration stress contributes to enhance plant defense response and mite performance on barley

M. Estrella Santamaria, Isabel Diaz, Manuel Martinez*

*** Correspondence:** Corresponding Author: m.martinez@upm.es

1 Supplementary Figures and Tables

1.1 Supplementary Figures

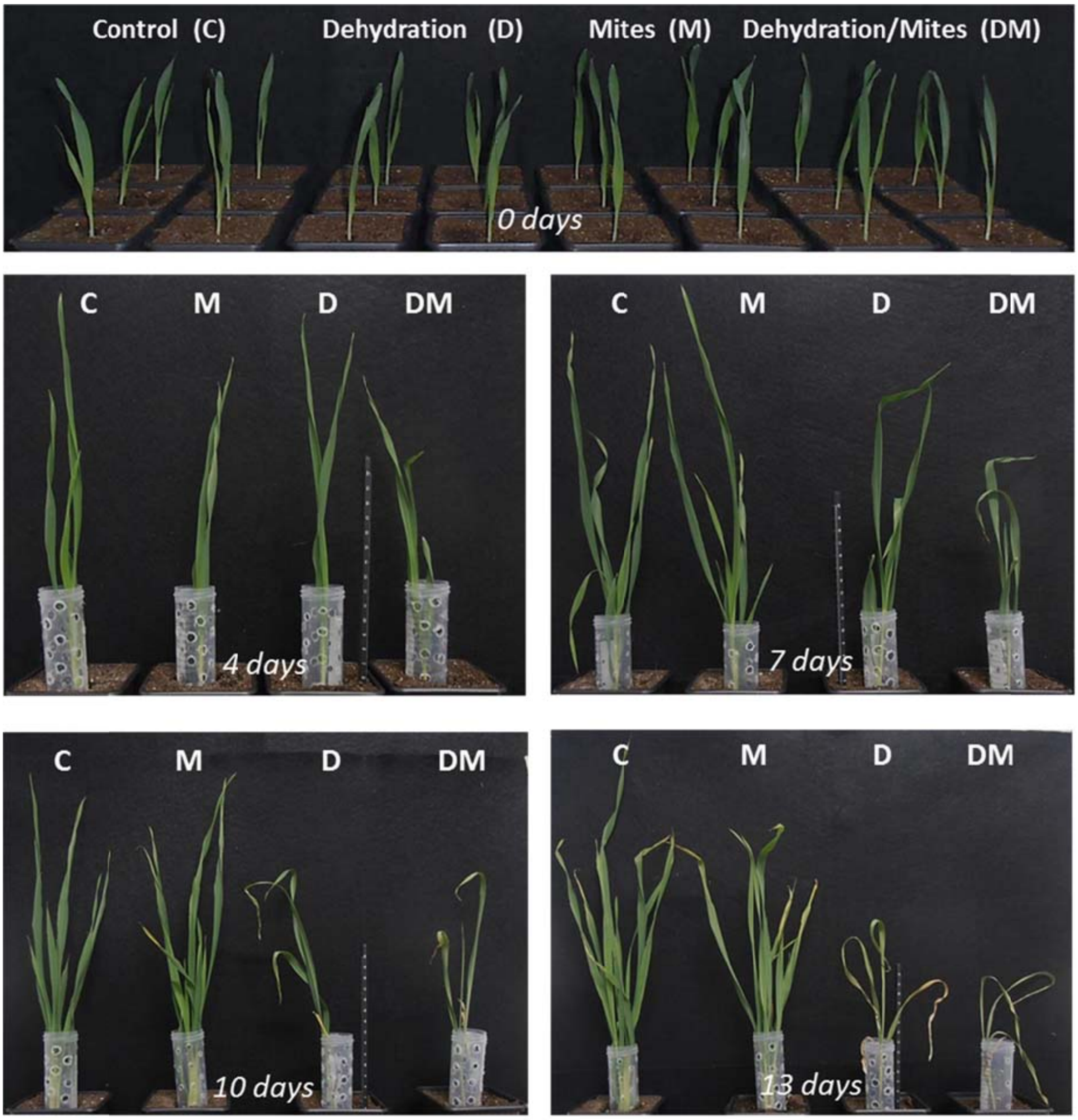

Supplementary Figure 1. Phenotype of barley plants 0, 4, 7, 10 and 13 days post treatment. Control (C), mites (M), dehydration (D) and dehydration + mites (DM).

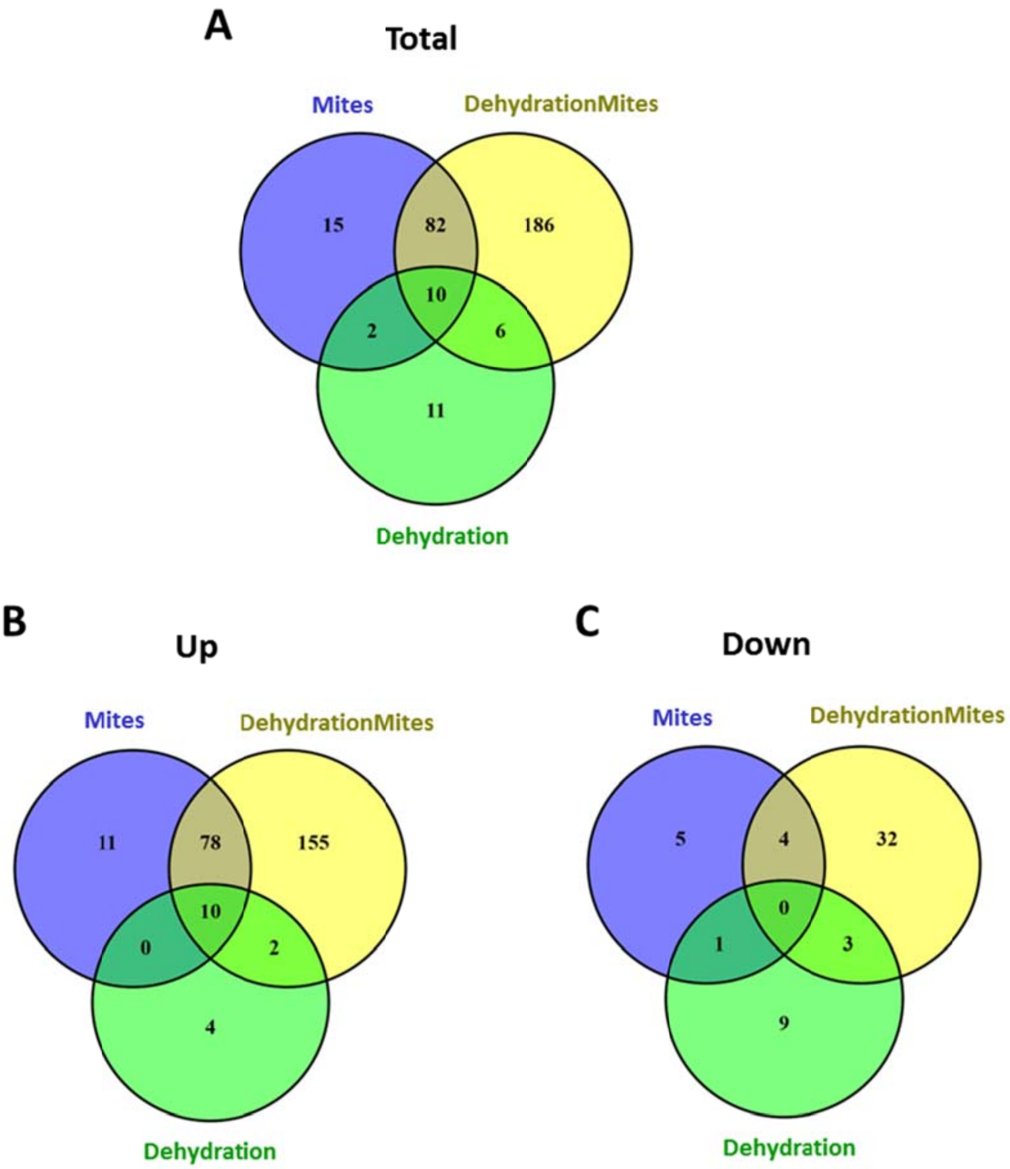

Supplementary Figure 2. Venn diagrams showing the number of specific and shared differential expressed genes among the different treatments and non-treated plants. **(A)** Total number of DEGs. **(B)** Up-regulated DEGs. **(C)** Down-regulated DEGs.

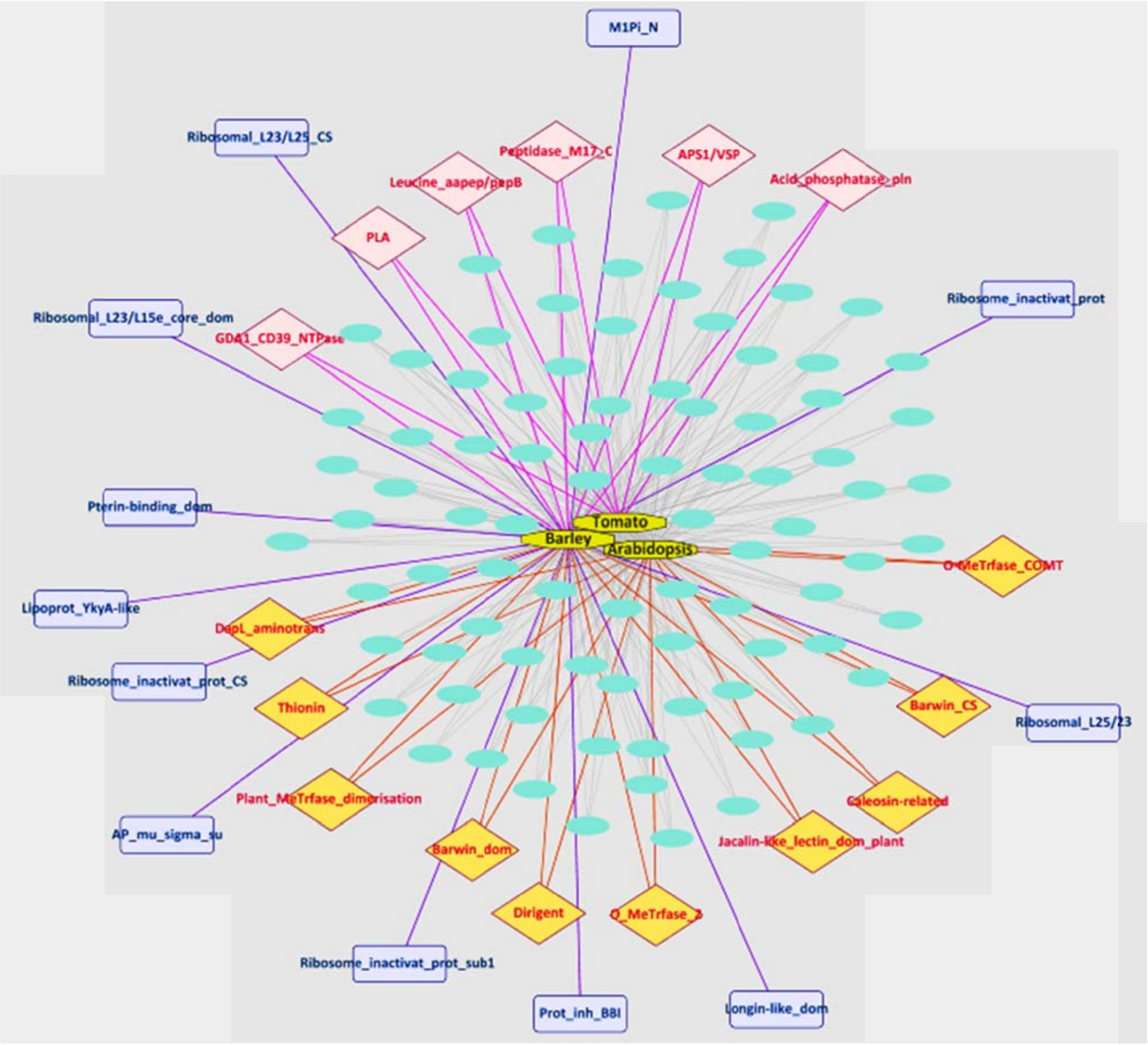

Supplementary Figure 3. Network showing the conservation of Interpro identifiers from DEGs after mites treatment in barley in the DEGs after mites treatment in tomato and Arabidopsis. Unique barley identifiers (purple), shared barley and tomato identifiers (pink), shared barley and Arabidopsis identifiers (orange), shared barley, tomato and Arabidopsis identifiers (blue-green).

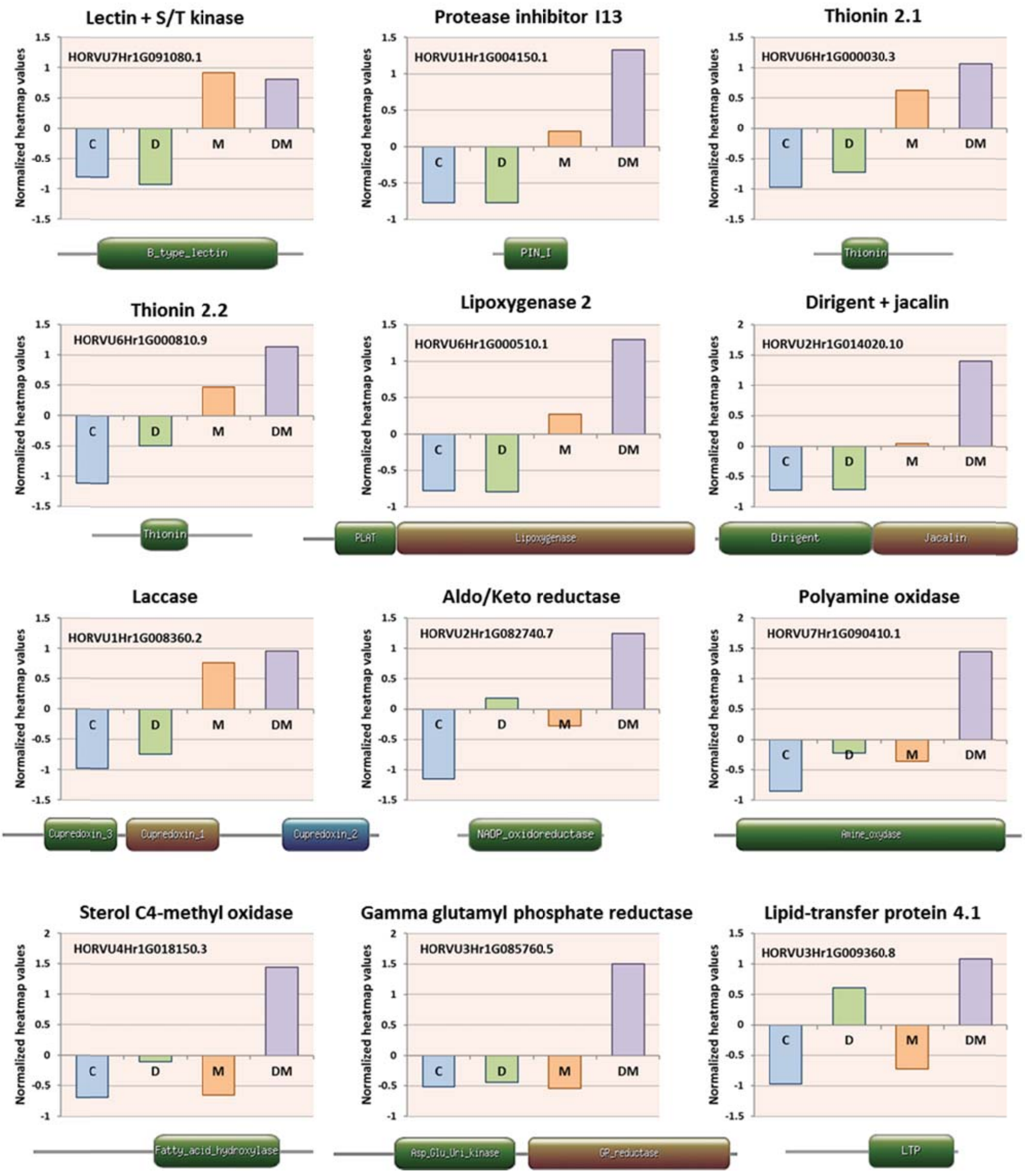

Supplementary Figure 4. RNA-seq expression of the selected DEGs after the different treatments. Values were normalized as heatmap values (see Figure 3). C, control. D, dehydration. M, mites. DM, dehydration and mites. A schematic representation of each gene showing the Interpro domains identified is located under the expression graphics.

1.2 Supplementary Tables

Supplementary Table 1. Primer sequences used for the amplification of barley genes by RT-qPCR assays.

Gene ID	Description	Primer name	Sequence (5´ → 3´)
Hv1Hr1G004150	Protease inhibitor I13	Hv1Hr1G004150-F	TCAAGGACATGCCTGAAGTG
		Hv1Hr1G004150-R	CGACAGTGTCAACGAGGATG
Hv1Hr1G008360	Laccase	Hv1Hr1G008360-F	AGGCCCACTACAACAACGTC
		Hv1Hr1G008360-R	TCGACTTGGTCGTGTACTGC
Hv2Hr1G014020	Jasmonate-induced protein (Dirigent + jacalin)	Hv2Hr1G014020-F	GCCGCGTTTGTAGAGATCA
		Hv2Hr1G014020-R	CCAGTAGTGGCATTGGAGTCT
Hv2Hr1G082740	Aldo/Keto reductase	Hv2Hr1G082740-F	GACATCTACGGTCCCCACAC
		Hv2Hr1G082740-R	GTGGCCAACTGGACCTTCT
Hv3Hr1G009360	Lipid transfer protein 4.1	Hv3Hr1G009360-F	CAGAGCACCGCTGACAATC
		Hv3Hr1G009360-R	TTAGAGCAGTCGACGGAAGC
Hv3Hr1G085760	Gamma glutamyl phosphate reductase	Hv3Hr1G085760-F	GCTCTAGCAATCCGAAGTGG
		Hv3Hr1G085760-R	CAACACCATCTGGAATCACG
Hv4Hr1G018150	Sterol C4-methyl oxidase	Hv4Hr1G018150-F	CCAGTACCTGCGTTCTGACA
		Hv4Hr1G018150-R	GTAGTCACCACCACGCCTTT
Hv6Hr1G000030	Thionin 2.1	HvThionin 2.1-F	GACGGGCAGAAACTGCTACAAC
		HvThionin 2.1-R	ACCGGATTCAGGGAGAAGAT
Hv6Hr1G000510	Lipoxygenase 2	Hv6Hr1G000510-F	AGAAGGGCGAAATGTACGTG
		Hv6Hr1G000510-R	CACCTTCAAGCCAGTGCATA
Hv6Hr1G000810	Thionin 2.2	HvThionin 2.2-F	TCAATCCAACATAGCCATTTC
		HvThionin 2.2-R	CAACTTTTTCCCTCCACCTG
Hv7Hr1G090410	Polyamine oxidase	Hv7Hr1G090410-F	GTACAAGCTGCAGCCCAAG
		Hv7Hr1G090410-R	GATGATGAGCAGCGAGACC
Hv7Hr1G091080	Lectin + Serine/threonine-protein kinase	Hv7Hr1G091080-F	AGCCACAGACATACGTGCAG
		Hv7Hr1G091080-R	GCGAGAGTATCGCCTTTTGT
Hv6Hr1G012570	Cyclophilin	Hv6Hr1G012570-F	TCCACCGGAGAGGAAGTACAGT
		Hv6Hr1G012570-R	AATGTGCTCAGAGATGCAAGGA
tetur18g03590	RP49	tetur18g03590-F	CTTCAAGCGGCATCAGAGC
		tetur18g03590-R	CGCATCTGACCCTTGAACTTC
tetur43g00010	VTG	tetur43g00010-F	ACGTCGCTATTGGTGAAACC
		tetur43g00010-R	TGTTGATTCGGGATGGGTAT
tetur03g03240	ATG13	tetur03g03240-F	ATGGCGGTGATAAGCTATGC
		tetur03g03240-R	AAGAAGCACCTGGCGAAGTA

Supplementary Table 2. List of the top ten significant results obtained from the enrichment gene ontology (GO) analysis of the Biological Process assigned to the DEGs.

		**Control/Mites**				
	**GO.ID**	**Term**	**Annotated**	**Significant**	**Expected**	**classicFisher**
**1**	GO:0006952	defense response	143	11	0.41	1.40E-13
**2**	GO:0006950	response to stress	694	13	1.99	2.90E-08
**3**	GO:0050896	response to stimulus	1171	13	3.36	1.20E-05
**4**	GO:0009617	response to bacterium	5	2	0.01	8.00E-05
**5**	GO:0042742	defense response to bacterium	5	2	0.01	8.00E-05
**6**	GO:0009620	response to fungus	7	2	0.02	0.00017
**7**	GO:0050832	defense response to fungus	7	2	0.02	0.00017
**8**	GO:0043207	response to external biotic stimulus	10	2	0.03	0.00036
**9**	GO:0051707	response to other organism	10	2	0.03	0.00036
**10**	GO:0098542	defense response to other organism	10	2	0.03	0.00036
		**Control/Dehydration and mites**				
	**GO.ID**	**Term**	**Annotated**	**Significant**	**Expected**	**classicFisher**
**1**	GO:0006952	defense response	143	14	1.02	1.20E-12
**2**	GO:0006950	response to stress	694	19	4.93	3.00E-07
**3**	GO:0044710	single-organism metabolic process	3207	45	22.78	3.60E-07
**4**	GO:0043207	response to external biotic stimulus	10	4	0.07	4.80E-07
**5**	GO:0051707	response to other organism	10	4	0.07	4.80E-07
**6**	GO:0098542	defense response to other organism	10	4	0.07	4.80E-07
**7**	GO:0009605	response to external stimulus	18	4	0.13	6.70E-06
**8**	GO:0009607	response to biotic stimulus	30	4	0.21	5.70E-05
**9**	GO:0055114	oxidation-reduction process	1986	29	14.11	6.70E-05
**10**	GO:0002682	regulation of immune system process	3	2	0.02	0.00015
		**Control/Dehydration**				
	**GO.ID**	**Term**	**Annotated**	**Significant**	**Expected**	**classicFisher**
**1**	GO:0006952	defense response	143	4	0.11	3.20E-06
**2**	GO:0006950	response to stress	694	4	0.55	0.0015
**3**	GO:0050896	response to stimulus	1171	4	0.93	0.0101

Mites/Dehydration and mites						
	**GO.ID**	**Term**	**Annotated**	**Significant**	**Expected**	**classicFisher**
**1**	GO:0001101	response to acid chemical	20	2	0.01	5.10E-05
**2**	GO:0009415	response to water	20	2	0.01	5.10E-05
**3**	GO:0010035	response to inorganic substance	31	2	0.02	0.00012
**4**	GO:1901700	response to oxygen-containing compound	31	2	0.02	0.00012
**5**	GO:0009628	response to abiotic stimulus	37	2	0.02	0.00018
**6**	GO:0006560	proline metabolic process	6	1	0	0.00335
**7**	GO:0006561	proline biosynthetic process	6	1	0	0.00335
**8**	GO:0006950	response to stress	694	3	0.39	0.005
**9**	GO:0042221	response to chemical	213	2	0.12	0.00571
**10**	GO:0046394	carboxylic acid biosynthetic process	218	2	0.12	0.00597
**Dehydration/Dehydration and mites**						
	**GO.ID**	**Term**	**Annotated**	**Significant**	**Expected**	**classicFisher**
**1**	GO:0043207	response to external biotic stimulus	10	3	0.03	4.40E-06
**2**	GO:0051707	response to other organism	10	3	0.03	4.40E-06
**3**	GO:0098542	defense response to other organism	10	3	0.03	4.40E-06
**4**	GO:0044710	single-organism metabolic process	3207	24	11	2.40E-05
**5**	GO:0009605	response to external stimulus	18	3	0.06	3.00E-05
**6**	GO:0002682	regulation of immune system process	3	2	0.01	3.40E-05
**7**	GO:0002831	regulation of response to biotic stimulus	3	2	0.01	3.40E-05
**8**	GO:0009627	systemic acquired resistance	3	2	0.01	3.40E-05
**9**	GO:0009814	defense response, incompatible interaction	3	2	0.01	3.40E-05
**10**	GO:0010112	regulation of systemic acquired resistance	3	2	0.01	3.40E-05
**Mites/Dehydration**						
	**GO.ID**	**Term**	**Annotated**	**Significant**	**Expected**	**classicFisher**
**1**	GO:0043207	response to external biotic stimulus	10	3	0.03	2.80E-06
**2**	GO:0051707	response to other organism	10	3	0.03	2.80E-06
**3**	GO:0098542	defense response to other organism	10	3	0.03	2.80E-06
**4**	GO:0009605	response to external stimulus	18	3	0.05	1.90E-05
**5**	GO:0006952	defense response	143	5	0.42	5.90E-05
**6**	GO:0009617	response to bacterium	5	2	0.01	8.40E-05
**7**	GO:0042742	defense response to bacterium	5	2	0.01	8.40E-05
**8**	GO:0009607	response to biotic stimulus	30	3	0.09	9.10E-05
**9**	GO:0006950	response to stress	694	9	2.05	0.00014
**10**	GO:0009620	response to fungus	7	2	0.02	0.00018
